# Sensitivity of Molds From Spoiled Dairy Products Towards Bioprotective Lactic Acid Bacteria Cultures

**DOI:** 10.3389/fmicb.2021.631730

**Published:** 2021-02-10

**Authors:** Ce Shi, Susanne Knøchel

**Affiliations:** Laboratory of Food Microbiology and Fermentation, Department of Food Science, University of Copenhagen, Copenhagen, Denmark

**Keywords:** bioprotection, spoilage molds, microbial interaction, antifungal activity, dairy products, manganese depletion

## Abstract

Fungal spoilage of dairy products is a major concern due to food waste and economical losses, some fungal metabolites may furthermore have adverse effects on human health. The use of lactic acid bacteria (LAB) is emerging as a potential clean label alternative to chemical preservatives. Here, our aim was to characterize the growth potential at three storage temperatures (5, 16, and 25°C) of a panel of molds (four *Mucor* and nine *Penicillium* strains) isolated from dairy products, then investigate the susceptibility of the molds toward 12 LAB cultures. Fungal cell growth and morphology in malt extract broth was monitored using oCelloScope at 25°C for 24 h. *Mucor plumbeus* 01180036 was the fastest growing and *Penicillium roqueforti* ISI4 (*P. roqueforti* ISI4) the slowest of the tested molds. On yogurt-agar plates, all molds grew at 5, 16, and 25°C in a temperature-dependent manner with *Mucor* strains growing faster than *Penicillium* strains regardless of temperature. The sensitivity toward 12 LAB cultures was tested using high-throughput overlay method and here all the molds except *P. roqueforti* ISI4 were strongly inhibited. The antifungal action of these LAB was confirmed when spotting mold spores on agar plates containing live cells of the LAB strains. However, if cells were removed from the fermentates, the inhibitory effects decreased markedly. The antifungal effects of volatiles tested in a plate-on-plate system without direct contact between mold and LAB culture media were modest. Some LAB binary combinations improved the antifungal activity against the growth of several molds beyond that of single cultures in yogurt serum. The role of competitive exclusion due to manganese depletion was examined as a possible antifungal mechanism for six *Penicillium* and two *Mucor* strains. It was shown that this mechanism was a major inhibition factor for the molds tested apart from the non-inhibited *P. roqueforti* ISI4 since addition of manganese with increasing concentrations of up to 0.1 mM resulted in partly or fully restored mold growth in yogurt. These findings help to understand the parameters influencing the mold spoilage of dairy products and the interactions between the contaminating strains, substrate, and bioprotective LAB cultures.

## Introduction

Mold spoilage is a major concern in dairy industry, resulting in significant food waste and substantial economic losses (Schnürer and Magnusson, 2005). Dairy products provide a favorable niche for mold growth, and visible alterations can be found due to the outgrowth on the surface of products. Some of the most common molds, such as *Penicillium* and *Mucor* genera, are involved in dairy product spoilage (Garnier et al., 2017). *Penicillium* spp. are mainly isolated from cheese but they are also found in other product types including yogurt, butter, and milk. *Mucor* spp. are common spoilage agents of yogurt and cheese (Pitt and Hocking, 2009). Apart from the negative impacts on food quality, such as visible growth and off-flavors, some fungal genera possess the ability to produce mycotoxins, which constitute a threat to human health. For instance, *Mucor circinelloides* (*M. circinelloides*) is recognized as an opportunistic pathogen, which can produce the mycotoxin 3-nitropropionic acid (Hollmann et al., 2008). It has been suspected of causing nausea, vomiting and diarrhea, and even a rhinocerebral mucormycosis in an immunosuppressed person, following ingestion of contaminated yogurt (Lazar et al., 2014). Several *Penicillium* strains are also known to be capable of producing mycotoxins, including wild-type *Penicillium roqueforti* (*P. roqueforti*) strains (Pitt and Hocking, 2009). Prevention of mold spoilage is therefore a vital part of the quality assurance in dairy industry.

To avoid fungal spoilage in food industry, much effort is used to prevent contamination and inhibit fungal growth during product manufacturing. Chemical preservatives have been used in food industry to protect food from undesirable growth and thus extend product shelf-life (Garnier et al., 2017), but there is a growing public concern about extensive use of chemical preservatives. Furthermore, an increasing number of molds are becoming resistant not only to antibiotics, but also to food preservatives, such as sorbic acid and benzoic acids (Schnürer and Magnusson, 2005). In recent years, there has been a rising demand from consumers for minimally processed or preservative-free food, which has resulted in a growing interest in natural fungal control alternatives, such as antifungal bioprotective cultures (Luz et al., 2018).

Selected lactic acid bacteria (LAB) have commonly been reported as potential biopreservation tools to inhibit fungal growth in different foodstuffs, extend shelf life, and sometimes even improve the nutritional value and sensory characteristics (Pelyuntha et al., 2019). For example, *Lactobacillus rhamnosus* (*L. rhamnosus*) was shown to have growth inhibitory effect on *Penicillium aurantioviolaceum* (*P. aurantioviolaceum*) and *Mucor plumbeus* (*M. plumbeus*) (Bazukyan et al., 2018) and *Lactobacillus paracasei* (*L. paracasei*) DGCC 2132 displayed antifungal properties against *Penicillium solitum* (*P. solitum*) DCS 302 and *Penicillium* sp. nov. DCS 1541 in yogurt (Aunsbjerg et al., 2015a). The mechanism underlying the activity of LAB strains against spoilage molds has been suggested to be multifactorial and include the production of a variety of metabolites including volatile compounds and non-volatile compounds (Campana et al., 2019). Leyva Salas et al. (2019) highlighted the diversity of antifungal molecules produced by bioprotective cultures in different dairy products but the concentrations of the individual compounds found in products have not been able to fully explain the inhibitive effect. Recently, Siedler et al. (2020) showed that competition for manganese is a major limiting factor for the growth of several spoilage organisms in yogurt when grown with a bioprotective culture consisting of *L. paracasei* and *L. rhamnosus* as the antifungal effect could be counteracted with the addition of manganese in increasing concentrations of up to 6 mg/L.

The aim of the present study was to: (a) characterize the growth potential at three temperatures of a panel of molds isolated from freshly fermented dairy products such as yogurt or skyr; (b) investigate the susceptibility of the molds toward different preparations of LAB by assessment of the direct interactive effect of molds with the LAB fermentates with or without living cells, as well as no direct contact assessing the role of volatiles; (c) evaluate the inhibitory effects of 12 LAB cultures in selected combinations on the growth of these molds and (d) investigate the role of competitive exclusion due to manganese depletion in inhibition.

## Materials and Methods

### Strains and Chemicals

The 12 LAB strains used in this study are listed in Table 1. The stock cultures were kept at −80°C in De Man, Rogosa and Sharpe (MRS) broth containing 20% glycerol. Thirteen molds isolated from freshly fermented dairy products were used as target organisms (Table 1). Malt extract agar (MEA, 30 g/L malt extract, 5 g/L peptone, 15 g/L agar, pH 5.6 ± 0.2) and malt extract broth (MEB, 17 g/L malt extract, 3 g/L peptone, pH 5.6 ± 0.2) were used to support fungal growth. Molds were grown on MEA plates for 7 days at 25°C, and the spore suspension of each mold was collected in sterile 0.9% saline solution (pH 7.0) and counted with a Malassez counting chamber. Suspension concentration of each mold was adjusted to 1.0 × 10^6^ spores/mL and stored at −80°C until further use. Manganese chloride (MnCl_2_) was obtained from Sigma-Aldrich (Schnelldorf, Germany) and used for the evaluation of manganese depletion. Commercial plain yogurt (3.5% fat, no sugar or additives, pH 4.6) was purchased from the local supermarket (Arla Foods, Copenhagen, Denmark).

**TABLE 1:** Microbial strains used in this study.

**Microbial strain**	**Abbreviation**	**Source**	**Provider**
**Bacteria**			
*Lacticaseibacillus rhamnosus* LRH01	*L. rhamnosus* LRH01	Dairy	SACCO
*Lacticaseibacillus rhamnosus* LRH05	*L. rhamnosus* LRH05	Dairy	SACCO
*Lacticaseibacillus rhamnosus* LRH14	*L. rhamnosus* LRH14	Dairy	SACCO
*Lacticaseibacillus rhamnosus* LRH16	*L. rhamnosus* LRH16	Cereals	SACCO
*Lacticaseibacillus rhamnosus* LRH43	*L. rhamnosus* LRH43	Dairy	SACCO
*Lactiplantibacillus plantarum* LP01	*L. plantarum* LP01	Dairy	SACCO
*Lactiplantibacillus plantarum* LP37	*L. plantarum* LP37	Cereals	SACCO
*Lactiplantibacillus plantarum* LP48	*L. plantarum* LP48	Meat	SACCO
*Lentilactobacillus parabuchneri* LPB02	*L. parabuchneri* LPB02	Dairy	SACCO
*Lentilactobacillus parabuchneri* LPB04	*L. parabuchneri* LPB04	Dairy	SACCO
*Lacticaseibacillus paracasei* LPC44	*L. paracasei* LPC44	Dairy	SACCO
*Lacticaseibacillus paracasei* LPC46	*L. paracasei* LPC46	Dairy	SACCO
**Fungi**			
*Mucor circinelloides* 01180023	*M. circinelloides* 01180023	Yogurt/Skyr	Arla
*Mucor plumbeus* 01180036	*M. plumbeus* 01180036	Yogurt/Skyr	Arla
*Mucor plumbeus* 01180037	*M. plumbeus* 01180037	Yogurt/Skyr	Arla
*Mucor plumbeus* 01180010	*M. plumbeus* 01180010	Yogurt/Skyr	Arla
*Penicillium commune* ISI2	*P. commune* ISI2	Greek yogurt	ISI
*Penicillium commune* 01180002	*P. commune* 01180002	Yogurt/Skyr	Arla
*Penicillium commune* 01180014	*P. commune* 01180014	Yogurt/Skyr	Arla
*Penicillium commune* 01180015	*P. commune* 01180015	Yogurt/Skyr	Arla
*Penicillium crustosum* 01180001	*P. crustosum* 01180001	Yogurt/Skyr	Arla
*Penicillium glabrum* ISI3	*P. glabrum* ISI3	Crème fraiche 18%	ISI
*Penicillium palitans* PPa01	*P. palitans* PPa01	Rahka (finnish quark)	SACCO
*Penicillium solitum* ISI5	*P. solitum* ISI5	Crème fraiche 30%	ISI
*Penicillium roqueforti* ISI4	*P. roqueforti* ISI4	Crème fraiche 18%	ISI

*All the bacteria were obtained from SACCO S.r.l (Italy) and incubated in MRS broth/agar at 37°C; the mold strains provided by Arla Foods (Denmark), ISI Food Protection (Denmark) and SACCO S.r.l. (Italy), respectively, were isolated from freshly fermented dairy products and incubated in MEA/MEB at 25°C. The names of all the bacteria are based on the taxonomy described by Zheng et al. (2020).*

### Growth Potential Assessment of 13 Dairy-Associated Molds

#### Growth Curves of 13 Molds Monitored by oCelloScope

Mold cell growth was monitored at 25°C for 24 h as described by Aunsbjerg et al. (2015b) using the oCelloScope detection system (BioSense Solutions ApS, Farum, Denmark), a newly developed optical detection system based on a unique optical scanning technology FluidScope^TM^ combining optical techniques. One hundred microliters of spore suspension of each mold (1.0 × 10^3^ spores/mL) in MEB medium were added into a 96-well microplate (approximately 100 spores/well). The 96-well microplate was standing at room temperature for around 1 h after inoculation to allow the spores to settle in these wells, and then placed in the oCelloScope system. The samples were measured automatically and the instrument-derived growth values were recorded every 60 min for 24 h. Automated growth kinetic analyses were performed using the Background Corrected Absorption (BCA) algorithm {BCA = log10[Σ(corrected absorption pixel histograms)]} of the oCelloScope-specific software, UniExplorer (v.5.0.3). For each mold strain, growth testing was performed with three technical replicates.

#### Mold Growth Potential on Yogurt-Agar Plates at Different Temperatures

Mold growth potential in plain yogurt was assessed by spotting 20 μL of spore suspension of each mold (1.0 × 10^5^ spores/mL) on yogurt-agar plates (yogurt was tempered in a 48°C water bath and then mixed with melted, tempered agar) in triplicate (2.0 × 10^3^ spores/spot) (Aunsbjerg et al., 2015a). Plates were incubated at 5, 16, and 25°C, respectively, for up to 24 days, to evaluate the effect of temperature on fungal growth. Multispectral images of plates with spotted molds were captured by a Videometer Lab2 spectral imaging instrument (Videometer ApS, Hørsholm, Denmark), and mold growth was expressed by the average size of three colonies in pixel units calculated according to Ebrahimi et al. (2015) using MATLAB 2018b software (MathWorks, inc., Natick, MA, United States).

### Sensitivity of Molds Toward 12 LAB Cultures in MRS

#### High-Throughput Overlay Test

**FIGURE 1:**
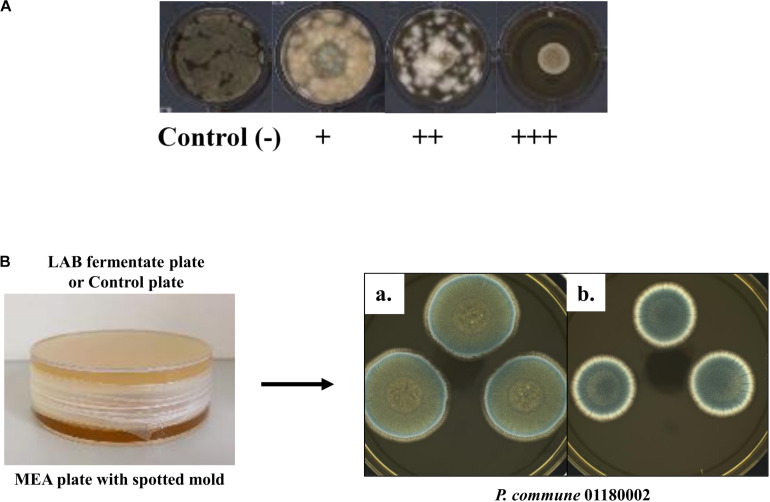
**(A)** Example of the antifungal inhibition scoring of LAB cultures overlaid by 0.5% malt extract soft agar containing *P. roqueforti* ISI4. Control: MRS agar in the absence of antifungal culture overlaid by molds; “+++”: strong inhibition; “++”: weak inhibition; “+”: growth but delayed sporulation; “–”: no inhibition. **(B)** A plate-on-plate system. Inhibitory effect of volatile compounds produced by *L. parabuchneri* LPB02 (plate on top) on growth of *P. commune* 01180002 on a MEA plate (bottom). Growth of *P. commune* 01180002 on the bottom MEA plate with a MRS fermentate of *L. parabuchneri* LPB02 plate on top **(b)** and a control plate on top **(a)** in a closed chamber, respectively, was assessed.

The antifungal activity of 12 LAB cultures against 13 molds was initially tested using the high-throughput overlay method developed by Garnier et al. (2018). The test was performed in 24-well microplates (Sigma-Aldrich) containing 500 μL of 1.5% MRS agar. One microliter of each culture suspension (1.0 × 10^7^ CFU/mL) was spotted on the center of each well and the 24-well microplates were incubated at 37°C for 48 h under anaerobic conditions. Thereafter, wells were overlaid with 100 μL of 0.5% malt extract soft agar inoculated with the individual mold at a final concentration of 1.0 × 10^3^ spores/mL (100 spores/well). Wells inoculated with molds but without LAB cultures were used as control. The 24-well microplates were incubated for up to 8 days at 25°C. The antifungal activity was determined by visually evaluating fungal growth as strong inhibition (+++), weak inhibition (++), fungal growth with delayed sporulation (+), and no inhibition (−) on the basis of the inhibition zone (Figure 1A). For each mold strain, growth inhibition testing was performed with three well replicates.

#### Sensitivity of the Molds Toward Different Preparations of LAB

In this test, the sensitivity of the target molds toward different preparations of LAB in MRS was evaluated by assessing the direct interactive effect of molds with the LAB fermentates containing live cells, with the LAB fermentates without cells, and with no direct contact between the molds and the LAB culture medium. MRS broth was inoculated with individual 12 LAB cultures at a final concentration of 1.0 × 10^7^ CFU/mL in 250 mL blue cap flasks and then fermented at 37°C for 22 h, which was regarded as sufficient to display antifungal activity (Aunsbjerg et al., 2015a), in order to obtain LAB fermentates. The pH and the number of CFU/mL of 12 LAB cultures were determined before and after fermentation, respectively. Cell-free fermentates were prepared by centrifugation of LAB fermentates with cells at 4, 400 × *g* for 20 min at 4°C, followed by filtration through a 0.45 μm filter (Syringe Filter Q-Max, Frisenette ApS, Denmark). Un-inoculated MRS broth kept at 37°C for 22 h was used as control. The antifungal activity of 12 LAB fermentates with or without live cells against target molds was measured using an agar spot method, and the contribution of volatile compounds produced by LAB to the antifungal activity was assessed in a “Plate-on-Plate” test system (Figure 1B) without direct contact between molds and LAB fermentates with cells (Aunsbjerg et al., 2015a).

##### Interaction of molds with LAB fermentates containing live cells

The agar plates (1% agar) of each LAB fermentate with bacterial cells were prepared by mixing with melted, tempered MEA (in a 48°C water bath). These agar plates were used after solidification and a short drying period. The antifungal activity of each LAB culture was tested by spotting 20 μL of each spore suspension (1.0 × 10^5^ spores/mL) on these LAB fermentates agar plates in triplicate and incubating at 25°C for 5 days. Control groups were prepared by spotting each mold suspension on the plates prepared by mixing un-inoculated MRS broth with MEA. Multispectral images of plates with spotted molds were captured by Videometer Lab2 spectral imaging instrument and the size of each mold colony was calculated based on the number of pixels using MATLAB 2018b software (Ebrahimi et al., 2015). The inhibitory effect was calculated according to the formula:

InhibitoryEffect(%)=(P-controlP)sample/P×control100

*P* indicates the total number of pixels of each colony in either control or sample groups.

##### Interaction of molds with cell-free LAB fermentates

The tests were performed as described above using the individual cell-free fermentates for the agar plates.

##### Interaction of molds with volatiles

An MEA plate was spotted with 20 μL of each mold spore suspension (1.0 × 10^5^ spores/mL) in triplicate. On top of the MEA plate, a plate with LAB fermentate agar containing live cells was placed upside down and the two plates were sealed together with parafilm. Control plates were prepared by mixing MRS broth with agar. The inhibitory effect of volatiles produced by bacterial cells on mold growth was assessed after 5 days at 25°C as described above.

Based on the results of antifungal activity of the 12 LAB culture, *Lactiplantibacillus plantarum* LP37 was selected as one representative of the most inhibitive cultures and used as the main antifungal culture in the test of antifungal activity of LAB binary combinations. According to the provider of the strain, the addition of the strain to a mild yogurt culture did not negatively affect the sensory attributes during a shelf life of 42 days at 10°C.

#### Antifungal Activity of LAB Binary Combinations in Yogurt Serum

Yogurt serum, which was used as a transparent yogurt surrogate in this assay, was prepared by centrifugation of the commercial plain yogurt for 30 min at 4, 400 × *g* (4°C) and subsequent filtration of the supernatant through a 0.22-μm pore size filter for sterilization (Syringe Filter Q-Max, Frisenette ApS, Denmark). The serum was kept at 4°C prior to use.

The antifungal activity of LAB binary combinations in yogurt serum was tested by pre-fermentation of LAB cultures either alone or in combination (Aunsbjerg et al., 2015c). Here, *L. plantarum* LP37 was selected as the main culture and combined with each of the other eleven cultures. Yogurt serum was inoculated with the single or combined cultures (in the ratio of 1:1) with the initial inoculum level of 10^5^ CFU/mL, and then fermented at 37°C for 22 h. After fermentation, the agar plates were prepared by mixing the fermentates of single or combined cultures, respectively, with melted, tempered agar. Un-inoculated yogurt serum agar plates (1% agar) were used as control. The inhibitory effect of LAB binary combinations and each LAB single culture was assessed by spotting spore suspension with 20 μL of 1.0 × 10^5^ spores/mL of each mold on these fermentate-agar plates, respectively. The four *Mucor* strains were incubated at 25°C for 2 days due to their rapid growth while the nine *Penicillium* strains were incubated for 5 days.

### Manganese Depletion as a Possible Antifungal Mechanism

In this test, *L. plantarum* LP37 was selected as a representative, inhibitive strain for exploring the role of manganese depletion in the growth inhibition of six *Penicillium* strains and two *Mucor* strains, representing different species of spoilage strains. The commercial plain yogurt was inoculated with *L. plantarum* LP37 at a final concentration of 1.0 × 10^7^ CFU/mL, and then distributed into four bottles, which were subsequently supplemented with manganese ion at the final concentration of 0, 0.001, 0.01, and 0.1 mM, respectively (Siedler et al., 2020). Yogurt without *L. plantarum* LP37 and manganese was used as control. The yogurt agar plates were prepared by mixing yogurt samples with melted, tempered agar. Then 20 μL of spore suspension (1.0 × 10^4^ spores/mL) of each mold were spotted on these yogurt agar plates in triplicate. After incubation for 5 days at 25°C, quantification of mold growth was performed as described in Subsection “Sensitivity of the Molds Toward Different Preparations of LAB.”

In addition, manganese depletion test was carried out in yogurt serum as well after pre-fermentation with *L. plantarum* LP37. In brief, *L. plantarum* LP37 was inoculated in yogurt serum at a final concentration of 1.0 × 10^7^ CFU/mL and then fermented at 37°C for 22 h prior to use. Both *L. plantarum* LP37 fermentates with or without bacterial cells were used for testing the manganese depletion. Manganese ion was added into yogurt serum as described above and the agar plates were prepared by mixing with agar. The pH and number of CFU/mL of *L. plantarum* LP37 were determined before and after fermentation. Un-inoculated yogurt serum kept at 37°C for 22 h was used as control. Each spore suspension was spotted as described above.

### Statistical Analyses

All the tests in this study were done in triplicate and the values were expressed as mean values ± standard deviation (SD). The statistical significances of experimental antifungal activity of LAB combinations were evaluated by one-way analysis of variance (ANOVA), differences were considered statistically significant at *P* < 0.05 (^∗^*P* < 0.05, ^∗∗^*P* < 0.01). Statistical analyses were performed using SPSS software ver. 26.0 (IBM, Armonk, NY, United States).

## Results

### Real-Time Monitoring of Cell Growth Using oCelloScope

Growth kinetics analysis of 13 molds during 24 h at 25°C in MEB medium (Supplementary Figure 1A) showed that the initial inoculum size of all the tested molds was approximately at the same level (100 spores/well), while large variations were observed in the growth potential. The *Mucor* strains generally grew faster than the *Penicillium* strains with *M. plumbeus* 01180036 as the most rapidly growing isolate. *M. circinelloides* 01180023 was the first isolate to reach stationary phase after approximately 10 h but reached a lower maximum level than the other strains. The fastest growing isolate among *Penicillium* strains was *Penicillium glabrum* ISI3, which grew almost as fast as the *M. plumbeus* strains. In contrast, *P. roqueforti* ISI4 cells displayed the slowest initial growth with respect to other strains tested. In addition, the morphological changes of molds, such as hyphae development, could also be studied visually from the oCelloScope images and quantified using morphological descriptors (Aunsbjerg et al., 2015b) as exemplified in Supplementary Figure 1B, showing the growth of *P. solitum* ISI5 and *M. circinelloides* 01180023 in MEB medium. Here, hyphae formation could be observed in the *Mucor* strain already within 4 h.

### Mold Growth Potential on Yogurt-Agar Plates

**FIGURE 2:**
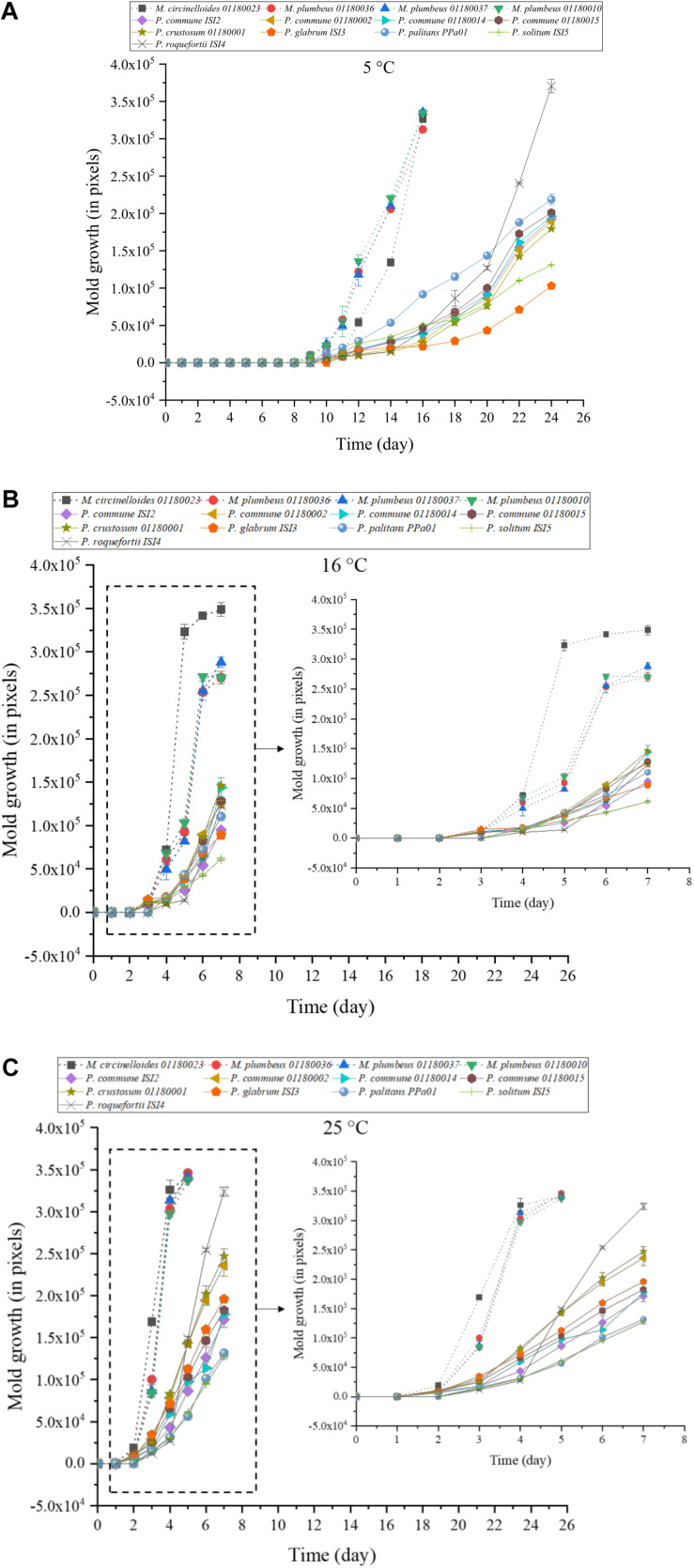
Growth potential of 13 molds on yogurt-agar plates incubated for up to 24 days at 5°C **(A)**, 16°C **(B)**, and 25°C **(C)**, respectively. The test was determined by spotting each spore suspension (20 μL of 1.0 × 10^5^ spores/mL) on yogurt-agar plates in triplicate. Bars represent the standard error of mean of three replicates. Four *Mucor* strains are marked with dotted lines; nine *Penicillium* strains with solid lines. The growth curves in **(B,C)** in the dotted box are magnified.

At the three tested temperatures, 5, 16, and 25°C, all tested molds were capable of growth on yogurt agar plates (Figure 2) in a temperature-dependent manner. For example, *M. circinelloides* 01180023 required around 14 days to reach a specific colony size of 1.5 × 10^5^ (in pixels) at 5°C, while this took 4 days at 16°C and 3 days at 25°C. At 5°C (Figure 2A), mold growth with sporulation was observed after incubation ranging from 9 to 11 days. Large variations were observed with *Mucor* strains generally growing faster than the *Penicillium* strains, and with *M. circinelloides* 01180023 as the fastest growing isolate. Growth of *P. roqueforti* ISI4 started out slowly, as also seen in the 24 h oCelloScope test, but it increased rapidly after some days and this strain had the highest growth recorded of the *Penicillium* tested after day 20 at 5°C and day 5 at 25°C, respectively. Also at 16°C (Figure 2B), the growth rate of *Mucor* strains was higher than *Penicillium* strains, and *M. circinelloides* 01180023 was still the faster growing mold. The results at 25°C (Figure 2C) were in accordance with the results in oCelloScope test. The four *Mucor* strains grew faster both in MEB medium and yogurt than the *Penicillium* strains regardless of temperature. Noteworthy, *P. glabrum* ISI3 grew rapidly at 25°C but it was the slowest isolate at 5°C, indicating that this strain is more sensitive to chilling. Moreover, *P. roqueforti* ISI4, initially the slowest growing mold, became the fastest growing *Penicillium* strain after incubation for 4 days. The mean values of the size of mold colonies were shown in Supplementary Table 1, and the values differed significantly (*P* < 0.05).

### Sensitivity Toward 12 LAB Cultures

#### High-Throughput Overlay Method

When employing a high-throughput overlay screening technique to detect antifungal activity of 12 LAB cultures against 13 molds, results obtained after 5 days were similar to those after 8 days of incubation. In this test, all tested molds were greatly inhibited (+++) by all the 12 LAB cultures at the concentration of 10^7^ CFU/mL except *P. roqueforti* ISI4, which showed resistance to all the tested LAB cultures except for a weak inhibition when exposed to *Lentilactobacillus parabuchneri* LPB04 (Table 2). The sporulation of this isolate was, however, delayed in all cases.

**TABLE 2:** Microbial strains used in this study.

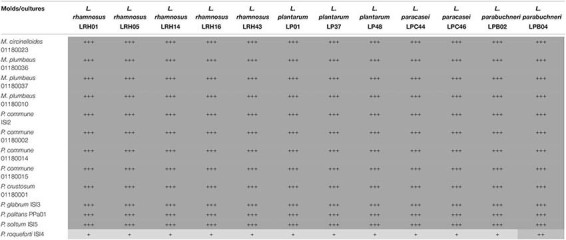

*The antifungal activity was scored as:*

*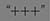: strong inhibition; 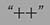: weak inhibition; 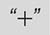: growth but delayed sporulation.*

#### Sensitivity Toward 12 LAB Fermentates With Live Cells

**FIGURE 3:**
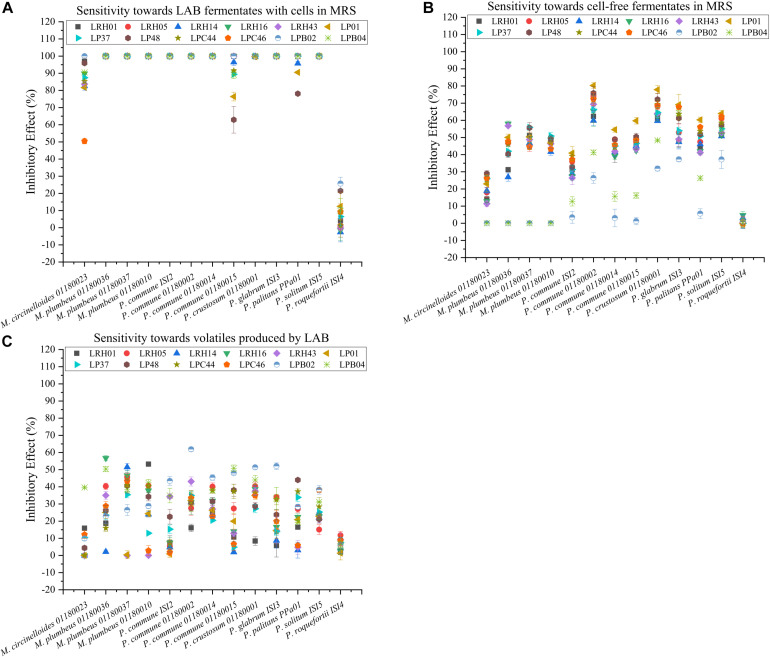
Sensitivity of 13 molds toward 12 LAB fermentates with live cells **(A)**, LAB cell-free fermentates **(B)**, and volatiles from LAB cultures in MRS **(C)**, respectively. The LAB strain code: LRH01, LRH05, LRH14, LRH16, and LRH43 are *L. rhamnosus* strains, LP01, LP37, and LP48 *L. plantarum* strains, LPC44 and LPC46 *L. paracasei* strains, and LPB02 and LPB04 *L. parabuchneri* strains. Bars represent the standard error of mean of three replicates.

The antifungal activity of 12 LAB fermentates with live cells in MRS against tested molds was tested. Results indicated that the inhibitory effect of the LAB fermentates with cells varied drastically (Figure 3A). In general, the 12 LAB cultures exhibited strong inhibition on the growth of all molds except *P. roqueforti* ISI4, which showed no or very weak inhibition as also seen previously in the high-throughput overlay test. In comparison with the results of the high-throughput overlay test, more differentiation of the response was observed since some strains (*M. circinelloides* 01180023, *Penicillium commune* 01180015 and *Penicillium palitans* PPa01) were only inhibited to a lesser degree (up to 50%). Comparing with other LAB cultures, *L. plantarum* LP48 showed weaker inhibition on *P. commune* 01180015 and *P. palitans* PPa01 with inhibitory effect of around 60 and 75%, respectively. The inhibitory effect of *L. paracasei* LPC46 on *M. circinelloides* 01180023 was only around 50%. Although some fermentates (e.g., *L. parabuchneri* LPB02 and *L. plantarum* LP48) displayed a slight inhibitory effect on *P. roqueforti* ISI4 of around 20–25%, these LAB cultures did not have the capacity to strongly inhibit the growth of this mold. After incubation for 9 days (data not shown), some tested molds were still strongly inhibited by the fermentates, while for other molds, some growth was observed after the 9 days, indicating a more fungistatic rather than fungicidal effect.

#### Sensitivity of the Molds to Cell-Free Fermentates

In this assay, the sensitivity of molds to LAB cell-free fermentates was determined in MRS medium. As seen in Figure 3B, the removal of cells resulted in decreased inhibition efficacy. Large variations of the inhibitory effect were seen with *P. commune* 01180002 being more sensitive, followed by *P. solitum* ISI5, whereas *P. roqueforti* ISI4 again was largely unaffected. Among the four LAB species tested, the *L. plantarum* cell-free fermentates gave rise to the strongest inhibitory effects whereas *L. parabuchneri* cell-free fermentates lost most of their effect. In addition, the antifungal compounds produced by *L. plantarum* LP01 displayed stronger inhibition on *P. commune* strains.

#### Sensitivity of the Molds to Volatiles Produced by Bacterial Cells

In order to test the antifungal volatiles, a plate-on-plate system was employed. Here, the inhibitory effect of volatile compounds produced by the 12 LAB cultures was modest, ranging from 0 to around 60% (Figure 3C). In contrast with the results in cell-free fermentates, the *L. parabuchneri* cultures here displayed higher inhibitory effect compared with other LAB cultures. The volatiles produced by *L. parabuchneri* LPB02 showed the highest inhibitory effects on *P. commune* 01180002 (60%), followed by *Penicillium crustosum* 01180001 and *P. glabrum* ISI3. Since the cell-free fermentate of the same strain, *L. parabuchneri* LPB02, gave rise to a relatively weak inhibition of these molds, this indicated that the activity of *L. parabuchneri* LPB02 grown in MRS against molds partly relied on volatiles.

### Inhibitory Effect of LAB Combinations in Yogurt Serum

Based on the results in MRS, *L. plantarum* LP37 was selected as a representative for the inhibitory cultures and used in binary combinations with each of the other eleven LAB cultures with an initial approximate inoculum level of 10^5^ CFU/mL. As shown in Table 3, 3 out of 11 binary combinations, including the binary combinations of *L. plantarum* LP37 combined with either *L. rhamnosus* LRH14, *L. plantarum* LP01, or *L. plantarum* LP48, showed significant improvement of antifungal activity against *P. commune* strains and/or *P. palitans* PPa01 compared with the corresponding single cultures (*P* < 0.05). *P. roqueforti* ISI4, which was neither inhibited by *L. plantarum* LP37 nor *L. paracasei* LPC46 on their own, was weakly inhibited by their combination, indicating some synergistic effect (Le Lay et al., 2016).

**TABLE 3:** Microbial strains used in this study.

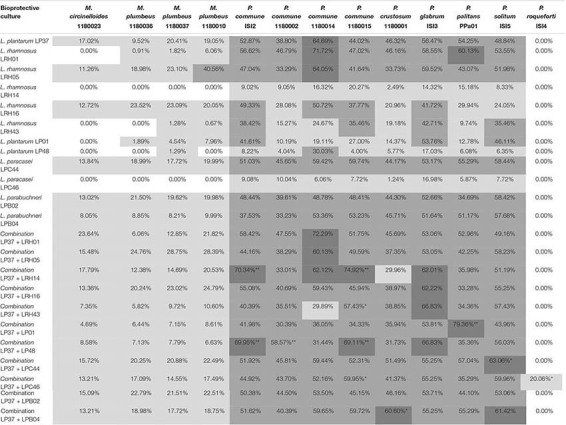

*The test was performed by spotting each spore suspension on agar plates prepared by mixing yogurt serum fermentate of each single LAB or combined culture with agar, then incubating for 2 days (four Mucor strains) or 5 days (nine Penicillium strains) at 25°C.*

*Inhibitory effect was calculated as described in Subsection “Sensitivity of the Molds Toward Different Preparations of LAB” based on the number of pixels of each colony.*

All the values in this table were expressed as the mean values of three replicates. The asterisk indicates significant difference between the treatment with binary combination cultures in comparison with the two corresponding single cultures (* P < 0.05, ** P < 0.01).

*Score of antifungal activity as follows:*

*“−” No inhibition, Inhibitory effect ≤ 0;*

*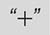 30% ≥ Inhibitory effect > 0;*

*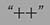 60% ≥ Inhibitory effect > 30%;*

*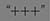 100% ≥ Inhibitory effect > 60%*.

### Manganese Depletion by *L. plantarum* LP37 in Yogurt and Yogurt Serum

**FIGURE 4:**
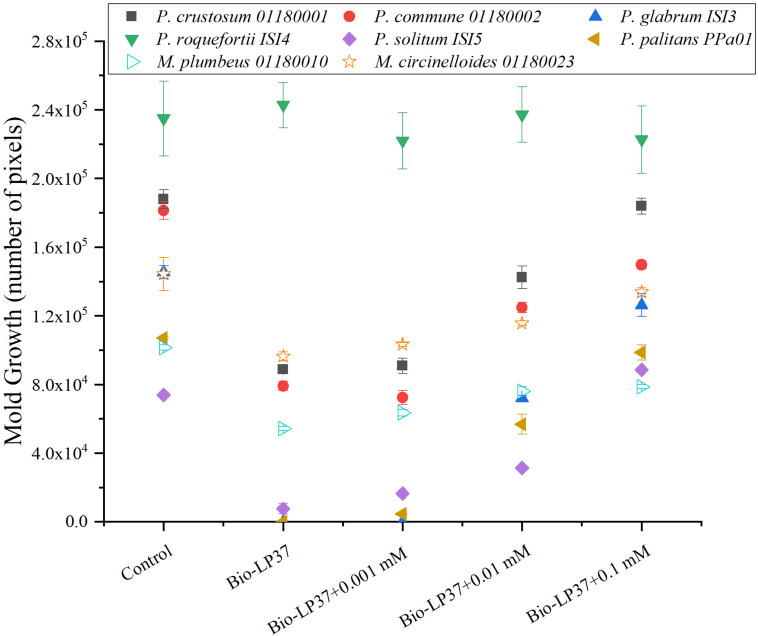
Manganese depletion by *L. plantarum* LP37 in yogurt. Different manganese concentrations (0.001, 0.01, and 0.1 mM) were added as indicated. The plates with spotted *Penicillium* strains and *Mucor* strains (200 spores/spot) were incubated at 25°C for 5 days. Bars represent the standard error of mean of three replicates. Bio-LP37 indicated the culture *L. plantarum* LP37. Closed symbols indicate *Penicillium* strains (6); Open symbols indicate *Mucor* strains (2).

In comparison with the control groups, the molds displayed various levels of sensitivity when grown with *L. plantarum* LP37 in yogurt-agar plates. However, the additions of increasing manganese concentrations (up to 0.1 mM) partly or fully restored the growth of the five sensitive *Penicillium* and two *Mucor* strains, suggesting that manganese depletion by *L. plantarum* LP37 plays a dominating role in the inhibition observed in these strains (Figure 4). Again, *P. roqueforti* ISI4 was almost non-affected by both *L. plantarum* LP37 and manganese.

**FIGURE 5:**
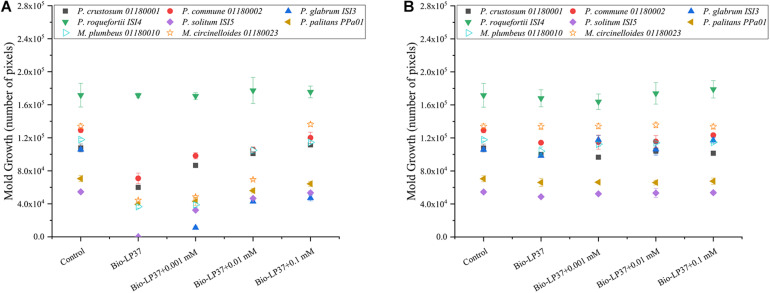
Manganese depletion by *L. plantarum* LP37 fermentate in yogurt serum with **(A)** or without bacterial cells **(B)**. Different manganese concentrations (0.001, 0.01, and 0.1 mM) were added as indicated. The plates with spotted *Penicillium* strains and *Mucor* strains (200 spores/spot) were incubated at 25°C for 5 days. Bio-LP37 indicates the bioprotective culture *L. plantarum* LP37. Closed symbols indicate six *Penicillium* strains; Open symbols indicate two *Mucor* strains.

In addition, yogurt serum was used as the culture medium to ferment LAB cultures. In Figure 5A, as expected, mold growth was partly or fully restored with addition of manganese in a concentration dependent manner, while *P. roqueforti* ISI4 was almost non-affected. Results here were in agreement with test carried out in yogurt, indicating that the manganese depletion of the culture medium was a limiting factor in both scenarios naturally low in manganese content. If cells were removed (Figure 5B), the antifungal activity of the cell-free yogurt serum was almost lost, indicating limited influence of any preformed compounds in the fermentate.

## Discussion

Different molds can spoil fresh, fermented dairy products but they may have different characteristics affecting their response. Here, the growth potential of *Penicillium* and *Mucor* species isolated from relevant products was determined as well as their response toward the activity of a range of LAB cultures. Firstly, the growth curves of molds were monitored in real time using the oCelloScope detection system, a small portable platform based on an automated optical detection system (Aunsbjerg et al., 2015b; Bouillon et al., 2019), during initial fungal growth at 25°C. The method is useful for early detection of growth before a three dimensional hyphae network has been formed (Aunsbjerg et al., 2015a). Growth was also followed on yogurt agar plates incubated at different temperatures, where 5°C was chosen to simulate a refrigeration storage temperature, 16°C an abuse cold chain temperature, and 25°C room temperature. Results from the oCelloScope test and the yogurt-agar spot test were in good agreement. However, the oCelloScope only covered the early growth. While *P. roqueforti* ISI4 grew slowly initially in both tests, the yogurt spot test, which ran for several days, revealed a later fast increase in the growth of this mold compared with the other *Penicillium* strains tested. The final high growth level supports Leggieri et al. (2017), who reported that *P. roqueforti* exhibited optimal growth at 25°C. Regardless of test method or temperatures, the growth of the *Mucor* strains seemed generally faster than the *Penicillium* strains. Similarly, Morin-Sardin et al. (2016) examined the growth of seven *Mucor* strains and found that they displayed strain differences in temperature optima, but they could, nevertheless, all be defined as fast. Gougouli et al. (2011) monitored mycelium growth rates of 12 mold strains grown in yogurt including six *Penicillium* spp. and one *M. circinelloides*, and found that although there were considerable differences within the *Penicillium* strains, the *M. circinelloides* strain still grew faster within a temperature range of 5–35°C. It has been suggested that the lack of septate formation in *Mucor* strains facilitates translocations within the cell and thereby promotes very rapid mycelium formation (Pitt and Hocking, 2009). All strains grew at 5°C underlining their potential for chilled storage spoilage. They did, however, grow slower on yogurt-agar than on MEA plates (Supplementary Figure 2). For example, the growth of *P. commune* 011800015 and *P. solitum* ISI5 with sporulation was observed after 7 days of incubation on MEA plates, whereas this took 10 and 9 days, respectively, on yogurt-agar plates. The differences could be partly due to the difference in pH as several previous studies such as Gock et al. (2003) and Morin-Sardin et al. (2016), who showed that the optimal pH for a variety of *Mucor* and *Penicillium* strains is closer to that of MEA (pH 5.6) rather than the pH 4.6 of yogurt.

The antifungal activity of 12 LAB cultures against the molds was screened in MRS medium using a high-throughput overlay method in 24-well microplates. The high-throughput antifungal overlay method allows for a rapid screening of a large variety of antifungal agents and the approach is feasible for many laboratories due to the ease and low equipment cost. In this system, all the LAB cultures had strong activity toward all the molds except *P. roqueforti* ISI4. The observed robustness of *P. roqueforti* toward a number of LAB cultures may be a common characteristics within this species, since this feature has also been observed in other *P. roqueforti* strains (Magnusson et al., 2003; Cheong et al., 2014; Russo et al., 2017; Quattrini et al., 2018). Although there is major intraspecific diversity in *P. roqueforti*, they are known to tolerate low concentrations of oxygen and high levels of CO_2_ or weak acid preservatives (Ropars et al., 2017; Coton et al., 2020). Using the overlay method, several studies have found different species of LAB to have strong antifungal activity. This includes Inglin et al. (2015), who reported both antifungal and antibacterial activity of LAB species; Garnier et al. (2018), who found that *Mucor racemosus* and *P. commune* could be inhibited by certain *L. rhamnosus* and *L. plantarum* cultures, and Salas et al. (2018), who found that a mixture of *Lactobacillus harbinensis* (*L. harbinensis*) and *L. plantarum* exhibited inhibitory effect on *Mucor* and *Penicillium* strains.

The sensitivity of these molds, apart from *P. roqueforti* ISI4, toward the 12 LAB cultures was confirmed in an agar spot test on fermentates with cells grown in MRS. This test, however, allowed for a slightly more differentiated response. When the cells were removed from the fermentates, the mold growth was still partly suppressed but the antifungal activity decreased markedly in comparison with that of fermentates with live cells. The remaining antifungal activity of cell-free fermentates in MRS is probably due to the presence of various active compounds produced by these LAB cultures in the supernatant such as organic acids, fatty acids, phenolic compounds, proteinaceous compounds and hydrogen peroxide (H_2_O_2_) which have been described as being antifungal (Dalié et al., 2010). Belguesmia et al. (2013) found that the cell-free culture supernatant of *L. harbinensis* in MRS had robust antifungal activities against *Penicillium expansum*, and observed that the supernatant contained high amounts of organic acids such as lactic and acetic acids as well as hexanoic acid. Guimarães et al. (2018) showed that the cell-free supernatants of *L. plantarum* and *Lactobacillus buchneri* inhibited *Penicillium nordicum* radial growth and suggested that this was due to the production of specific organic acids, such as lactic acid, phenyllactic acid, hydroxyphenyllactic acid and indole lactic acid. Apart from antifungal compounds in the cell-free fermentates, it is also possible that nutrient depletion has taken place although MRS is a rich medium. As reported by previous study, Honoré et al. (2016) indicated that glucose and glutamine in a chemically defined medium were almost fully consumed by *L. paracasei* strains, while other nutrients remained at 50% or more of the initial content, which was speculated to correlate with the decreased mold growth. In nature, competitive exclusion due to the competition for nutrients (Honoré et al., 2016), space (Golowczyc et al., 2007), and essential trace ions is a widespread phenomenon also between bacteria and fungi (Mille-Lindblom et al., 2006). Very recently, Siedler et al. (2020) has pointed toward manganese depletion as a major antifungal mechanism in a yogurt context. Compared with yogurt, MRS has a high amount of trace ions making depletion less likely to explain all the inhibition observed. The specific contribution of volatile metabolites produced by LAB cultures to the antifungal activity was estimated in a non-contact assay. Here, some inhibitory activity of volatile compounds was observed although the effect was much lower than that of cell-containing fermentates of LAB cultures in MRS. Our results here confirm that some of the bacterial volatiles (Fernando et al., 2005) have a measurable antifungal effect, albeit modest in this system. Leyva Salas et al. (2019) suggested that the inhibition of *P. commune* in sour cream was associated with higher amounts of three volatile compounds produced by LAB: diacetyl, acetoin and an unidentified volatile compound; Arrebola et al. (2010) likewise reported the antifungal properties of volatile compounds such as diacetyl, acetoin, dimethyl sulfone, 2-butanone, and volatile fatty acids and Aunsbjerg et al. (2015a) found that in a defined medium, the main volatile compound measured in the headspace of a cell-containing fermentate of *L. paracasei* during fermentation was diacetyl, which had a strong inhibitory effect on two *Penicillium* strains.

Although laboratory media optimized for growth of LAB cultures are well suited for an initial screening of the antifungal activity of the cultures, they do not reflect the composition of the relevant foods nor previous starter culture activity. Further investigations were therefore conducted in yogurt and yogurt serum in order to simulate the conditions in yogurt better. Manganese, an essential trace element, is a key cofactor for the growth of mold (Avila et al., 2013) and the level in milk is low (0.03 mg/L) (Siedler et al., 2020). According to Böhmer et al. (2013), due to the absence of superoxide dismutase, *L. plantarum* requires a relatively high level of manganese ions for optimal growth (up to 30 mM) under aerobic conditions. *L. plantarum* LP37 was selected as a representative of an inhibitory LAB and was capable of inhibiting growth of 5 out of 6 selected *Penicillium* spp. (*P. roqueforti* was unaffected) and two *Mucor* strains in yogurt and yogurt serum. In all cases of inhibition, the growth could be partly or fully restored by addition of manganese up to 0.1 mM, thus illustrating that not only the *Penicillium* but also the *Mucor* strains were inhibited by the lack of accessible manganese. The inhibition of mold growth in yogurt therefore seems to be associated with the ability of the *L. plantarum* culture to efficiently scavenge manganese, further supporting that in dairy products the removal of manganese accessible to molds is a main inhibitory mechanism of bioprotective cultures. This also implies that the same cultures may not have the same effect if the products are based on or mixed with materials with high manganese content such as plant materials.

In comparison with the inhibitory effect of the 12 LAB fermentates with live cells in MRS (Figure 3A), the sensitivity of these target molds decreased when the LAB fermentates were based on yogurt serum (Table 3). It was also observed that *L. plantarum* LP37 exhibited stronger antifungal activity against both *Mucor* and *Penicillium* strains in MRS medium than observed in yogurt or yogurt serum (Supplementary Figure 5). In the cell-free *L. plantarum* LP37 yogurt serum fermentate, mold growth was not inhibited (Figure 5B) in contrast to what was observed in the MRS based media (Figure 3B). The lack of inhibition may partly be due to the less optimal growth conditions for the LAB culture in yogurt serum, where both pH (pH 4.6) and nutrient levels are lower than in MRS (start pH 5.6). It was observed that in MRS medium, the number of bacterial cells of *L. plantarum* LP37 reached 10^9^ CFU/mL after the 22 h fermentation (Supplementary Figure 3), while only a level of approximately 10^8^ CFU/mL was reached in the yogurt serum (Supplementary Figure 4). Production of compounds with antifungal effect would therefore also be predicted to be present in much higher concentrations and thereby potentially plays a larger role in the MRS fermentates compared with the yogurt serum. This further illustrates the importance of testing in matrices relevant to the food in question when evaluating interactions. A broad panel of target organisms is also desirable since it showed that both *Penicillium* and *Mucor* species were sensitive to the manganese depletion, but also that some molds such as *P. roqueforti* are not affected and will require different countermeasures.

The enhancement of the inhibitory effect by employment of combinations of different LAB cultures or mixed with propionibacteria has been noted in several studies (Schwenninger and Meile, 2004; Salas et al., 2018) and several mixed bioprotective cultures are commercially available. The mixtures may have the advantage of employing different mechanisms or support mutual growth without having a negative impact on the sensory properties. The option of using different LAB combinations can therefore increase the technological flexibility in terms of products and target organisms and may also decrease costs, if it is possible to use lower inocula than seen for single cultures. Here, binary combinations containing *L. plantarum* LP37 were tested in yogurt serum to investigate potential synergistic effects. Results indicated that the binary combination of *L. plantarum* LP37 and *L. plantarum* LP48 in yogurt serum had significantly improved antifungal activity (*P* < 0.05) against some molds, two *P. commune* strains, compared with single cultures (Table 3). It is noteworthy that the inhibitory effects observed in mixed cultures were obtained with relatively low start inocula, which could make the use of bioprotective cultures more economically feasible. On the other hand, higher start inocula would most likely ensure stronger inhibitory effects.

Overall, the interaction between LAB and spoilage fungi is very complex and the LAB will exert antifungal activity in different modes depending on the circumstances making it challenging to develop all-purpose bioprotective cultures. On the other hand, selection of cultures for a particular environment such as plain yogurt or similar dairy products, can be facilitated by recognizing the role of manganese accumulation capacity in this environment. Further work should be undertaken to define which interaction mechanisms are at play in different scenarios and how factors such as oxygen and production of reactive oxygen species, as well as compositional changes of the matrix, influence the effect of the bioprotective cultures.

## Conclusion

Dairy products are very susceptible to spoilage molds, and it is therefore crucial to characterize the growth potential of molds. Here, *Mucor* strains grew faster than *Penicillium* strains both in MEB medium and yogurt. All tested molds, except *P. roqueforti* ISI4, were strongly inhibited by the 12 LAB strains when they were grown in MRS. After removal of the LAB living cells from MRS based fermentates, the inhibitory effect decreased, and the effect of volatiles alone was even less, indicating that preformed compounds played a role but they were not the main inhibitory factor. When yogurt serum was employed as a medium, a reduced LAB growth and a weaker inhibition of mold growth were observed, while removal of the cells caused almost complete loss of activity. The inhibition efficacy could in some instances be enhanced by combining cultures. In yogurt, it was shown that manganese depletion by *L. plantarum* LP37 played an important role in the growth inhibition of both the *Penicillium* and the *Mucor* strains tested. Apart from the non-affected *P. roqueforti* ISI4, it therefore seems that these spoilage molds are generally sensitive to bioprotective cultures capable of making the manganese unavailable in the matrix. Elucidation of the importance of growth conditions, manganese sources, and the rate of manganese transport would further help to expand the use of bioprotective cultures in similar matrices. The inhibitory effect and mode of action of the bioprotective cultures thus seems to depend not only on the target organisms, and growth of the culture but also on the composition of the matrix.

## Data Availability Statement

The raw data supporting the conclusions of this article will be made available by the authors, without undue reservation.

## Author Contributions

CS designed and performed the experiments, contributed to the conception and data analysis, and prepared the manuscript. SK designed and supervised the project, analyzed the data, and reviewed the manuscript. Both authors read and approved the final manuscript.

## Conflict of Interest

The authors declare that the research was conducted in the absence of any commercial or financial relationships that could be construed as a potential conflict of interest.
