# Supplementary material for: Sensitivity of Molds From Spoiled Dairy Products Towards Bioprotective Lactic Acid Bacteria Cultures
Source: Front Microbiol. 2021 Feb 10;12:631730. doi: 10.3389/fmicb.2021.631730 (PMC7902714; doi:10.3389/fmicb.2021.631730)
Supplement: Supplementary file 1 [file Data_Sheet_1.docx]

Supplementary Material


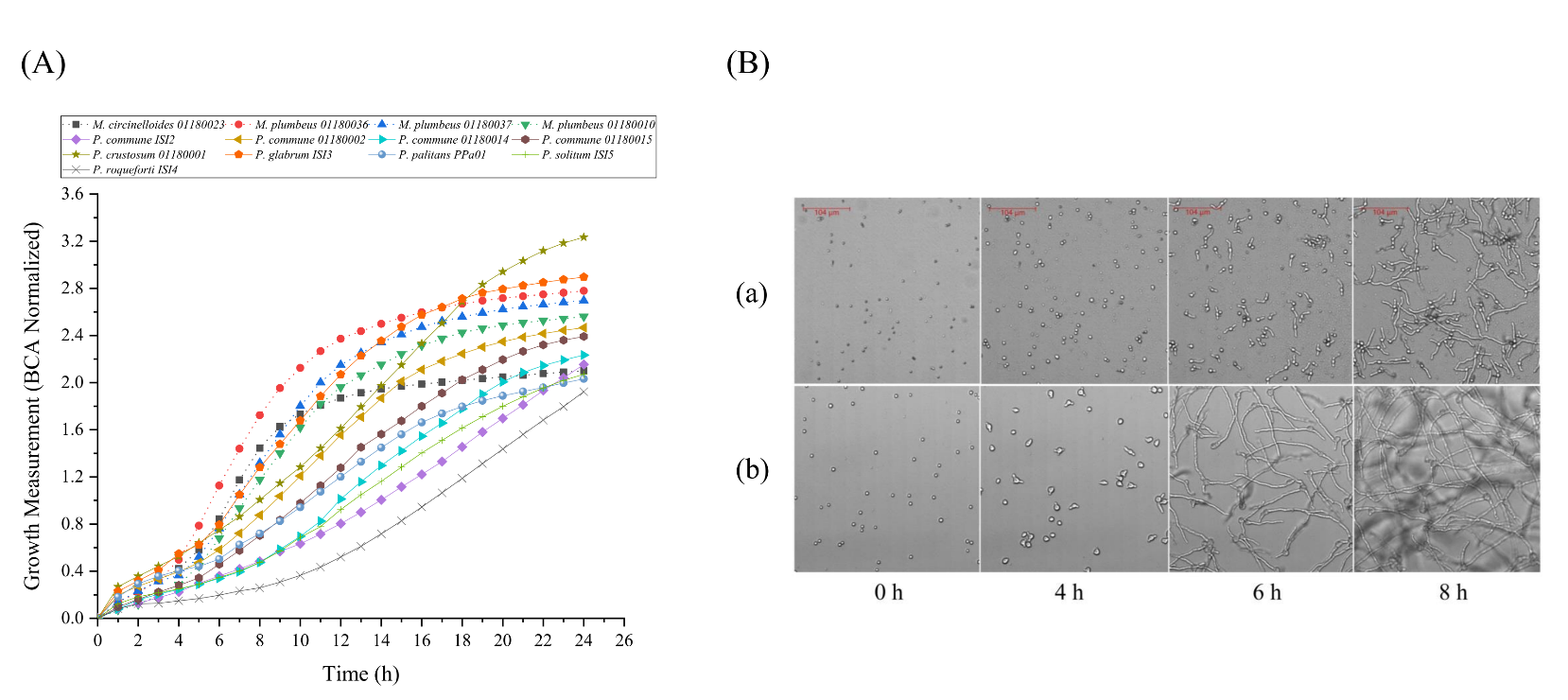


**Supplementary Figure 1.** (A) Growth kinetics analysis of 13 molds monitored by oCelloScope. Data were presented as the mean values of three replicates. One hundred microliter of each spore suspension (1.0 × 10^3^ spores/mL) in MEB was added to a 96-well microplate in triplicate and then incubated at 25 ℃ for 24 h. The growth curves of four *Mucor* strains were shown in dot line and nine *Penicillium* strains were shown in solid line. (B) oCelloScope images of *P. solitum* ISI5 (a) and *M. circinelloides* 01180023 (b) grown in MEB medium. Scale bar: 104 μm.

(A)

(B)

**Supplementary Figure 2.** Growth curves of 13 molds (four *Mucor* strains, dot line; nine *Penicillium* strains, solid line) determined by spotting each spore suspension (20 μL of 1.0 × 10^5^ spores/mL) on yoghurt-agar plates (A) and MEA plates (B), respectively, in triplicate at 5 ℃. Quantification of mold growth based on the size (the number of pixels) of each colony was calculated by MATLAB 2018b software. Bars represent the standard error of mean of three replicates.

(A)

(B)

**Supplementary Figure 3.** The changes of pH (A) and the number of CFU/mL (B) of 12 LAB cultures in MRS broth before and after fermentation for 22 h at 37 ℃. Bars represent the standard error of mean of three replicates. The dotted lines indicate the respective value of Y axis (A, pH value; B, Log_10_ CFU/mL).

(A)

(B)

**Supplementary Figure 4.** The changes of pH (A) and the number of CFU/mL of *L. plantarum* LP37 (B) in yoghurt serum before and after fermentation for 24 h at 37 ℃. Un-inoculated yoghurt serum served as Control. YS: yoghurt serum; LP37: *L. plantarum* LP37. Bars represent the standard error of mean of three replicates. The dotted lines indicate the values of Y axis (Log10 CFU/mL).

**Supplementary Figure 5.** Inhibitory effect of *L. plantarum* LP37 on six *Penicillium* strains and two *Mucor* strains in yoghurt, yoghurt serum and MRS medium, respectively. In yoghurt, *L. plantarum* LP37 was inoculated directly into yoghurt without fermentation; In yoghurt serum and MRS, *L. plantarum* LP37 was inoculated first and then fermented for 22 h at 37 ℃.

**Supplementary Table 1.** Colony size of 13 molds (in pixel unit) on yoghurt-agar plates incubated for up to 24 days at 5 ℃ (A), 16 ℃ (B) and 25 ℃ (C), respectively.

| (A) |  |  |  |  |  |  |  |  |  |  |  |  |  |
| --- | --- | --- | --- | --- | --- | --- | --- | --- | --- | --- | --- | --- | --- |
| Incubation time  (day) | *M.circinelloides*  01180023 | *M. plumbeus*  01180036 | *M. plumbeus*  01180037 | *M. plumbeus*  01180010 | *P. commune* ISI2 | *P. commune* 01180002 | *P. commune* 01180014 | *P. commune* 01180015 | *P. crustosum* 01180001 | *P. glabrum*  ISI3 | *P. palitans* PPa01 | *P. solitum*  ISI5 | *P. roqueforti* ISI4 |
| 8 | 0.00 | 0.00 | 0.00 | 0.00 | 0.00 | 0.00 | 0.00 | 0.00 | 0.00 | 0.00 | 0.00 | 0.00 | 0.00 |
| 9 | 0.00a | 10498.33b | 8489.00b | 9813.33b | 0.00a | 0.00a | 0.00a | 0.00a | 0.00a | 0.00a | 0.00a | 4903.33ab | 0.00a |
| 10 | 5972.00a | 21052.67c | 25401.33c | 23288.67c | 9511.00ab | 0.00a | 7108.00a | 3176.00ab | 0.00a | 0.00a | 14689.33bc | 6296.00ab | 6764.50ab |
| 11 | 11870.67a | 57773.67b | 49195.67b | 55394.00b | 12571.67a | 8486.00a | 9238.33a | 8482.67a | 7509.00a | 13205.33a | 20341.00a | 13468.33a | 9344.33a |
| 12 | 54154.00c | 121725.67de | 117980.33d | 135971.00e | 15846.67ab | 11977.00a | 18051.33ab | 17427.67ab | 9460.67a | 16482.67ab | 29099.33b | 25928.00ab | 10759.67a |
| 14 | 134330.67f | 205782.00g | 210031.67gh | 220128.67h | 27099.67bcd | 17513.67ab | 29137.33cd | 28033.00bcd | 15064.67a | 20140.33abc | 53463.33e | 34588.67d | 15004.33a |
| 16 | 326896.33f | 312394.33e | 335886.00f | 334382.67f | 39626.00b | 28951.33a | 38334.33b | 47010.33bc | 26066.67a | 21664.33a | 91609.00d | 50188.67c | 43093.33bc |
| 18 | ND | ND | ND | ND | 65561.67b | 61134.33b | 58059.67b | 67989.00b | 53820.00b | 29106.67a | 115251.33d | 58412.33b | 86222.00c |
| 20 | ND | ND | ND | ND | 91707.67d | 86057.67cd | 90867.00d | 100124.33e | 75949.67b | 43242.00a | 143372.00g | 78932.67bc | 126830.67f |
| 22 | ND | ND | ND | ND | 154572.00d | 151158.67d | 161367.67e | 172789.00f | 142168.33c | 71147.67a | 188018.33g | 110078.33b | 240328.67h |
| 24 | ND | ND | ND | ND | 192500.00de | 189016.00cd | 196155.00de | 201026.67e | 179415.00c | 103089.00a | 218862.00f | 130952.33b | 370459.00g |

| (B) |  |  |  |  |  |  |  |  |  |  |  |  |  |
| --- | --- | --- | --- | --- | --- | --- | --- | --- | --- | --- | --- | --- | --- |
| Incubation time  (day) | *M.circinelloides*  01180023 | *M. plumbeus*  01180036 | *M. plumbeus*  01180037 | *M. plumbeus*  01180010 | *P. commune* ISI2 | *P. commune* 01180002 | *P. commune* 01180014 | *P. commune* 01180015 | *P. crustosum* 01180001 | *P. glabrum*  ISI3 | *P. palitans* PPa01 | *P. solitum*  ISI5 | *P. roqueforti* ISI4 |
| 0 | 0.00 | 0.00 | 0.00 | 0.00 | 0.00 | 0.00 | 0.00 | 0.00 | 0.00 | 0.00 | 0.00 | 0.00 | 0.00 |
| 1 | 0.00 | 0.00 | 0.00 | 0.00 | 0.00 | 0.00 | 0.00 | 0.00 | 0.00 | 0.00 | 0.00 | 0.00 | 0.00 |
| 2 | 0.00 | 0.00 | 0.00 | 0.00 | 0.00 | 0.00 | 0.00 | 0.00 | 0.00 | 0.00 | 0.00 | 0.00 | 0.00 |
| 3 | 10744.00bc | 10480.67bc | 11009.67bc | 12257.33cd | 8859.00b | 10221.00bc | 11186.00bc | 9501.67bc | 11009.33bc | 14841.67d | 0.00a | 0.00a | 0.00a |
| 4 | 71984.00c | 60517.67bc | 49741.67b | 68430.33c | 15303.00a | 11393.67a | 16287.00a | 17167.67a | 11546.00a | 17734.67a | 16253.00a | 15878.00a | 9719.67a |
| 5 | 323014.00h | 92760.33f | 81838.33e | 103159.00g | 25384.00b | 43128.67d | 36955.67cd | 39294.33cd | 39860.67d | 38680.00cd | 43184.67d | 29557.33bc | 14081.67a |
| 6 | 341616.00h | 253633.00f | 255160.67f | 271738.33g | 53906.67ab | 89842.67e | 64156.00bc | 82140.00de | 86332.33e | 67471.67c | 72099.67cd | 42810.00a | 60732.33bc |
| 7 | 348936.67h | 270464.00f | 287889.33g | 270188.00f | 94870.67b | 125392.00d | 143873.33e | 128409.00d | 145786.00e | 89126.67b | 110353.00c | 61471.67a | 122875.33cd |

| (C) |  |  |  |  |  |  |  |  |  |  |  |  |  |
| --- | --- | --- | --- | --- | --- | --- | --- | --- | --- | --- | --- | --- | --- |
| Incubation time  (day) | *M.circinelloides*  01180023 | *M. plumbeus*  01180036 | *M. plumbeus*  01180037 | *M. plumbeus*  01180010 | *P. commune* ISI2 | *P. commune* 01180002 | *P. commune* 01180014 | *P. commune* 01180015 | *P. crustosum* 01180001 | *P. glabrum*  ISI3 | *P. palitans* PPa01 | *P. solitum*  ISI5 | *P. roqueforti* ISI4 |
| 0 | 0.00 | 0.00 | 0.00 | 0.00 | 0.00 | 0.00 | 0.00 | 0.00 | 0.00 | 0.00 | 0.00 | 0.00 | 0.00 |
| 1 | 0.00 | 0.00 | 0.00 | 0.00 | 0.00 | 0.00 | 0.00 | 0.00 | 0.00 | 0.00 | 0.00 | 0.00 | 0.00 |
| 2 | 18994.00d | 8533.00bc | 7987.00bc | 7952.00bc | 8479.67bc | 10295.67c | 6586.00bc | 10599.00c | 9356.00bc | 8196.33bc | 0.00a | 0.00a | 0.00a |
| 3 | 168970.00g | 100132.33f | 86624.67e | 84440.00e | 17708.67ab | 29507.00cd | 17606.67ab | 25091.00bc | 24927.67bc | 34529.00d | 15474.67a | 14178.00a | 11805.33a |
| 4 | 326259.67h | 303412.33fg | 313452.67g | 297326.00f | 43303.00b | 78379.67e | 59261.67c | 65081.00cd | 82754.33e | 71399.00de | 31678.67ab | 30755.00a | 27267.33a |
| 5 | 341742.33f | 346272.67f | 341352.67f | 337485.67f | 86537.67b | 143085.33e | 97435.33c | 102922.67c | 142521.00e | 112627.00d | 56625.00a | 60956.67a | 148772.33e |
| 6 | ND | ND | ND | ND | 126183.00c | 194324.33e | 113752.67bc | 146514.33d | 202208.00e | 159666.00d | 101333.33ab | 94997.67a | 254342.00f |
| 7 | ND | ND | ND | ND | 171465.33b | 235951.33d | 177816.67bc | 182697.33bc | 247083.33d | 195867.67c | 131588.00a | 127359.33a | 323896.67e |

Note: 1. The mean values (in pixel unit) which indicated the size of each mold colony in these tables were taken from three replicates.

2. The different letters indicated that the colony size of different mold strains grown for the same incubation time differed significantly (*P* < 0.05).

3. ND: not detection.
